# Correlation of CD47 Expression with Adverse Clinicopathologic Features and an Unfavorable Prognosis in Colorectal Adenocarcinoma

**DOI:** 10.3390/diagnostics11040668

**Published:** 2021-04-08

**Authors:** Hyunsung Kim, Seungyun Jee, Yeseul Kim, Jongmin Sim, Seongsik Bang, Hwang Kyu Son, Hosub Park, Jaekyung Myung, Young Hyeh Ko, Seung Sam Paik

**Affiliations:** 1 Department of Pathology, Hanyang University College of Medicine, Seoul 04763, Korea; hhnt5841@gmail.com (H.K.); Jee.seung.yun@gmail.com (S.J.); grypony@naver.com (S.B.); ganzi4900@gmail.com (H.K.S.); Parkhstm@gmail.com (H.P.); tontos016@naver.com (J.M.); 2202378@hyumc.com (Y.H.K.); 2 Department of Pathology, University of Ulsan College of Medicine, Asan Medical Center, Seoul 25440, Korea; coabee@hanmail.net; 3 Department of Pathology, Korea University College of Medicine, Seoul 02841, Korea; j07star2@gmail.com

**Keywords:** colorectal adenocarcinoma, CD47, immunohistochemistry, immune checkpoints, prognosis

## Abstract

CD47, a transmembrane protein, is widely overexpressed on the tumor cell surface. However, the prognostic significance of CD47 expression in colorectal adenocarcinoma (CRA) has not yet been clarified. Here, we investigated the clinicopathologic significance of CD47 expression in CRA. CD47 expression was evaluated via immunohistochemical analysis of microarray sections of 328 CRA tissues. CD47 expression was observed in 53 (16.2%) of the 328 CRA tissues, and positive expression was associated with lymphatic invasion (*p* = 0.018), perineural invasion (*p* = 0.024), tumor budding (*p* = 0.009), the pathologic N stage (*p* = 0.022), and the American Joint Committee on Cancer (AJCC) stage (*p* = 0.027). In survival analyses of 329 patients, a positive CD47 expression was associated with a poor recurrence-free survival (RFS) (*p* = 0.032). In multivariate analysis, however, it was not an independent prognostic factor. In patients who underwent surgical resection without adjuvant treatment, a positive CD47 expression was associated with a shorter RFS (*p* = 0.001) but not with cancer-specific survival (CSS). In patients who received postoperative adjuvant treatment, no significant differences were found in both RFS and CSS. In conclusion, we investigated CD47 expression in 328 CRA tissues. A positive CD47 expression was observed in a minority (16.2%) of the tissues and was significantly associated with adverse clinicopathologic features and a poor patient outcome.

## 1. Introduction

Colorectal adenocarcinoma (CRA) is the third most common malignancy and the second leading cause of cancer-related deaths worldwide [1]. Although clinical advances in early detection and curative surgery have led to five-year survival rates of 90% for localized CRA, the survival of patients with advanced CRA remains poor [2]. As personalized medicine evolves, an accurate prediction of prognosis has become essential for determining the appropriate treatment for cancer patients [3]. Although many candidate biomarkers have been investigated for the prediction of CRA prognosis and targeted therapy, the identification of effective biomarkers remains challenging [4]. Therefore, new, reliable, and practical biomarkers that can be used as prognostic factors and as candidates for targeted therapy are warranted.

Tumor development involves an interplay between cancer cells, stromal cells, and the immune system [5]. The balance between cancer cells and the immune system is disrupted during carcinogenesis, conferring the capacity of evasion from host immune elimination to cancer cells [5]. The inflammatory process plays an important role in the carcinogenesis of CRA [6]. Patients with inflammatory bowel disease, such as ulcerative colitis and Crohn’s disease, have an elevated risk of developing CRA, principally resulting from the pro-neoplastic effects of chronic inflammation [7]. Tumor-associated macrophage (TAM) involves the activation and elimination of cancer cells through phagocytosis [8]. There are emerging efforts to explore TAM as a target of immunotherapy [9]. A recently identified cell surface molecule that regulates the interaction between TAM and colon cancer cells is CD47 [10].

CD47 is a glycoprotein physiologically expressed on the surface of normal cells and acts as an immune checkpoint [11]. CD47 binds to signal regulator protein alpha (SIPRα), which is expressed on the surface of macrophages and inhibits phagocytosis [12]. CD47 overexpression has been reported in various types of malignancy and is associated with a poor prognosis in solid and hematologic malignancies, including non-small cell lung cancer, breast cancer, stomach cancer, and non-Hodgkin lymphoma [13,14,15,16]. As an anticancer treatment, anti-CD47 antibody has shown promising results in non-Hodgkin lymphoma, malignant pediatric brain tumor, lung cancer, and liver cancer [17,18,19,20,21]. Cancer immunotherapy targeting CD47 has demonstrated success at the preclinical level and is now under clinical investigation for various human malignancies [22]. However, CD47 expression in CRA is less well-characterized and its prognostic significance according to the microsatellite instability (MSI) status and treatment modalities has not been clarified.

We investigated the immunohistochemical expression of CD47 in 328 cases of CRA and analyzed the correlation between its expression and various clinicopathologic factors. Additionally, to reveal the prognostic significance of CD47, survival analyses were conducted according to MSI molecular subgroups and treatment modalities.

## 2. Materials and Methods

### 2.1. Patients and Tumor Samples

A total of 390 patients who were newly diagnosed and underwent curative surgery at Hanyang University Hospital (Seoul, Korea) between 2005 and 2010 were enrolled in this study. Patients who were diagnosed as recurrent, who had received neoadjuvant treatment, who were with incomplete clinical data, or who were without available paraffin blocks were excluded, resulting in a total of 328 patients. In addition, 48 samples of normal colorectal tissue were selected from 328 total cases to compare the CD 47 expression between normal and cancer tissues. Among 48 cases, three cases without qualified paraffin blocks were excluded, resulting in 45 normal samples. Two surgical pathologists (S. S. Paik and H. Kim) reviewed the hematoxylin and eosin (H&E)-stained slides and medical records to identify the clinicopathologic characteristics. A protocol for examining specimens from patients with primary adenocarcinoma of the colon and rectum (the College of American Pathologists) and the eighth American Joint Committee on Cancer (AJCC) TNM staging classification were used to determine the pathologic staging and other pathologic characteristics [23,24]. The pathologic characteristics included the tumor size, gross type (polypoid, ulcerofungating, and ulceroinfiltrative), histologic grade (G1: well; G2: moderate; G3: poor; G4: undifferentiated), lymphatic invasion, vascular invasion, perineural invasion, tumor deposit, tumor budding, the pathologic T (pT) stage, the pathologic N (pN) stage, extranodal tumor extension, distant metastasis, the AJCC stage, and MSI status [24,25]. Recurrence-free survival (RFS) was defined as the time interval between the surgical date and the date of disease progression, relapse, or death from CRA. Cancer-specific survival (CSS) was defined as the time interval between the date of surgery and the date of death. This study was approved by the Institutional Review Board of Hanyang University Hospital (HYUH 2019-11-008-002) and the requirement for informed consent was waived.

### 2.2. Tissue Microarray Construction

We used a manual tissue microarrayer (Unitma, Seoul, Korea) for tissue microarray (TMA) construction from archived formalin-fixed, paraffin-embedded tissue blocks. The representative area of the carcinoma (>0.5 cm) was selected from H&E-stained sections using light microscopy. Tissue cylinders (3 mm in diameter) were punched from a previously marked area on each donor block and transferred to the recipient block.

### 2.3. Microsatellite Analysis

Microsatellite analysis was performed using a panel of five microsatellite markers (BAT25, BAT26, D17S250, D2S123, and D5S346) according to the National Cancer Institute workshop-recommended consensus [26]. MSI-high was defined as a shift of microsatellites for two or more markers. MSI-low was defined as a shift of microsatellites for one marker. MSI-low and stable microsatellites were indicative of a non-MSI-high case.

### 2.4. Immunohistochemical Staining

Immunohistochemical staining of CD47 was performed with 4-µm-thick sections from TMA blocks using the Ventana Benchmark XT automated staining system (Ventana Medical Systems, Tucson, AZ, USA) according to the manufacturer’s protocol. Anti-CD47 rabbit monoclonal antibody (Abcam, Cambridge, UK, EPR21794) was used with a dilution factor of 1:200.

### 2.5. Interpretation of Immunohistochemical Staining

**Figure 1:**
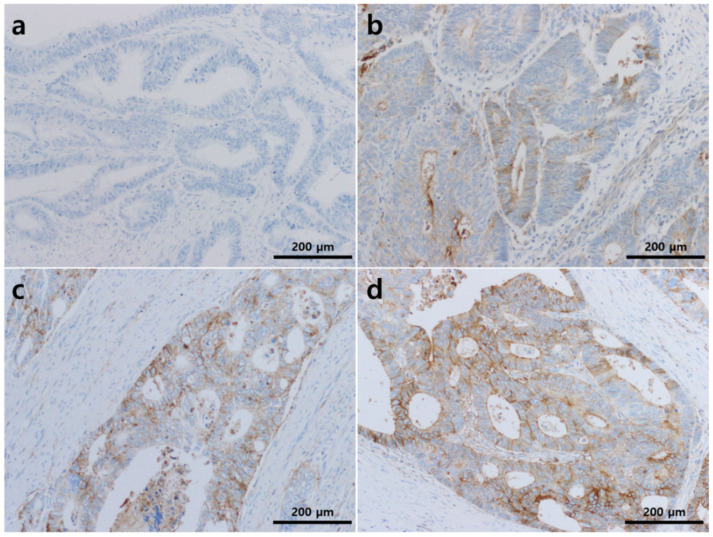
CD47 expression in colorectal adenocarcinoma tissues. The intensity of membranous staining was graded as (**a**) negative, 0; (**b**) weak, 1+; (**c**) moderate, 2+; or (**d**) strong, 3+ (**a**–**d**, ×200).

The membrane staining of tumor cells was evaluated using the H-score method. The percentage of stained tumor cells was reviewed and scored using a four-tier system (0, negative; 1+, weak; 2+, moderate; 3+, strong). Figure 1 shows representative microscopic images according to the intensity. The H-score was calculated as follows:

H-score = 1 × (% of 1+ cells) + 2 × (% of 2+ cells) + 3 × (% of 3+ cells).

The total immunoreactivity score ranged from 0 to 300. The cases were subdivided into negative expression (H-score < 50) and positive expression groups (H-score ≥ 50) using the receiver operating characteristic curve. All immunoreactivity assessments were done in a blinded fashion regarding the clinicopathologic factors and patient survival.

### 2.6. Statistical Analysis

Statistical analysis was performed using SPSS software version 21 (IBM Corp., Armonk, NY, USA). The chi-square test was used to assess the association between CD47 expression and clinicopathologic factors, including gross type, histologic grade, lymphatic invasion, vascular invasion, perineural invasion, tumor deposit, tumor budding, the pT stage, the pN stage, extranodal tumor extension, distant metastasis, the AJCC stage, and MSI status. Differences in CD47 immunoreactivity scores between normal and cancer tissues and differences in tumor size between positive and negative CD47 expression groups were compared using the Mann–Whitney U-test. The Kaplan–Meier method with a log-rank test and the Cox proportional hazards regression model were used for survival analyses. Statistical significance was set at *p* < 0.05.

## 3. Results

### 3.1. Baseline Characteristics of the Cohort

The clinicopathologic characteristics of the patients are summarized in Table 1. The median follow-up period was 127 months (range, 1–182), and the mean age at surgery was 63.6 ± 11.19 years. A total of 202 (61.6%) patients were men, with a male-to-female ratio of 1.6:1. Pathologic evaluation revealed that 17 cases (5.2%) were of histologic grade 1, 159 (48.5%) were of grade 2, 133 (40.5%) were of grade 3, and 19 (5.8%) were of grade 4. According to the eighth AJCC staging system, 36 (11.0%) were of stage I, 102 (31.1%) were of stage II, 167 (50.9%) were of stage III, and 23 (7.0%) were of stage IV. Among the 328 cases, 303 (92.4%) were non-MSI-high and 25 (7.6%) were MSI-high. Distant metastasis was identified in 23 (7.0%) patients at the time of initial diagnosis, and 73 patients (22.3%) had experienced relapse or metastasis during the follow-up period.

### 3.2. CD47 Expression in Normal and Cancer Tissues

We evaluated the CD47 expression in 45 samples of normal colorectal tissue and 328 samples of CRA tissue. The mean CD47 expression score was 2.22 (±7.03) in normal colorectal tissues and 19.20 (±45.04) in CRA tissues. The mean CD47 expression score was significantly higher in CRA tissues compared to normal colorectal tissues (*p* = 0.013, Mann–Whitney test).

### 3.3. CD47 Expression and Correlation with Clinicopathologic Features

A positive CD47 expression was not observed in normal colonic mucosal tissues (0%) but was observed in 53 CRA tissues (16.2%). Table 2 shows the association between CD47 expression and the clinicopathologic factors. In all cases, a positive CD47 expression was significantly correlated with lymphatic invasion (*p* = 0.018, chi-square test), perineural invasion (*p* = 0.024, chi-square test), tumor budding (*p* = 0.009, chi-square test), the pN stage (*p* = 0.022, chi-square test), and the AJCC stage (*p* = 0.027, chi-square test). The tumor size, gross type, histologic grade, vascular invasion, tumor deposit, the pT stage, extranodal tumor extension, distant metastasis, and MSI status were not associated with CD47 expression.

### 3.4. Prognostic Significance of CD47 Expression

**Figure 2:**
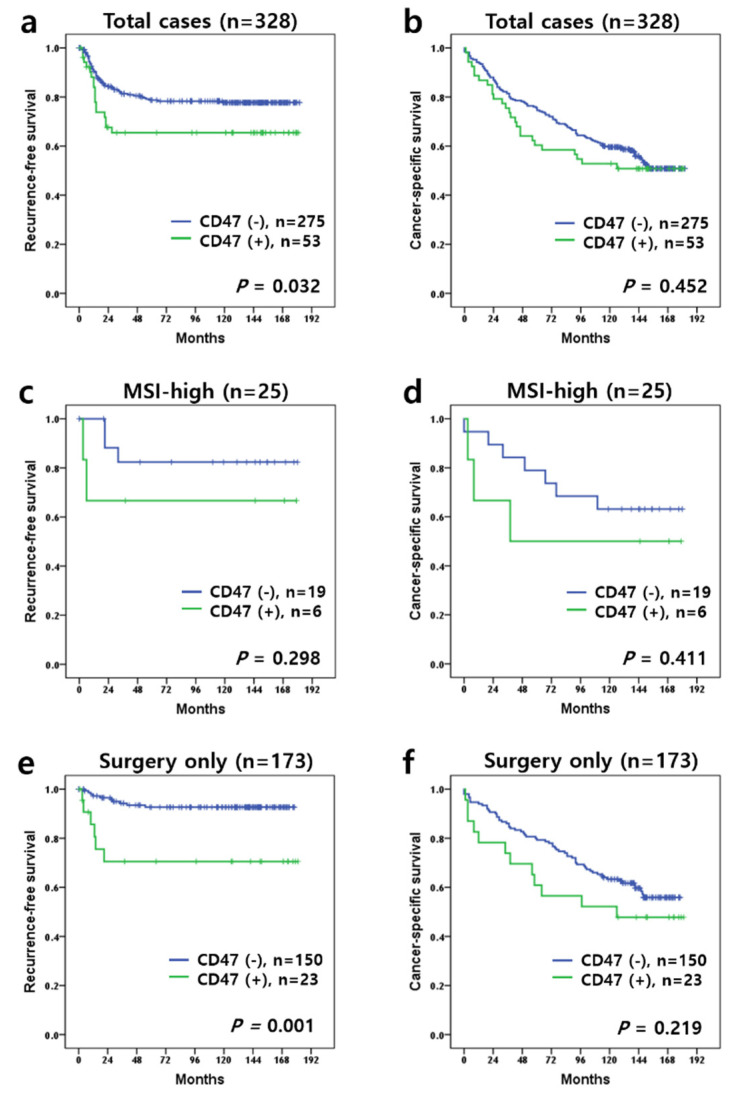
Kaplan–Meier survival curves of colorectal adenocarcinoma patients stratified based on CD47 expression. (**a**) Recurrence-free survival (RFS) and (**b**) cancer-specific survival (CSS) in all patients. (**c**) RFS and (**d**) CSS in MSI-high patients. (**e**) RFS and (**f**) CSS in patients who received surgical resection without adjuvant treatment.

In a total of 328 patients, patients with positive CD47 expression achieved significant shorter RFS lengths compared to those with negative CD47 expression (mean RFS, 84.4 months vs. 95.5 months; *p* = 0.032, log-rank test) (Figure 2a). There was no significant difference between the positive and negative CD47 expression groups regarding the CSS (*p* = 0.452, log-rank test) (Figure 2b). We additionally subdivided the groups according to MSI status and treatment modality. In the MSI subgroup, there was no significant difference in both RFS and CSS in the non-MSI-high group (*p* = 0.583 and *p* = 0.057, respectively, log-rank test) and in the MSI-high group (*p* = 0.298 and *p* = 0.411, respectively, log-rank test) (Figure 2c,d). In patients who received surgical resection only, a positive CD47 expression was associated with a shorter RFS (mean RFS, 88.0 months vs. 107.9 months; *p* = 0.001, log-rank test) (Figure 2e); however, a significant difference in CSS was not identified (*p* = 0.219, Figure 2f). In patients who received postoperative adjuvant treatment, no significant difference was found in both RFS and CSS (*p* = 0.995 and *p* = 0.819, respectively, log-rank test). Univariate Cox regression analysis revealed that the AJCC stage and treatment modality correlated with poor RFS, and the AJCC stage correlated with poor CSS (Table 3). Multivariate Cox regression analysis demonstrated that the treatment modality was an adverse prognostic factor for RFS, and the AJCC stage was a poor prognostic factor for CSS (Table 3). CD47 expression was associated with poor RFS based on univariate analysis (*p* = 0.035); however, it was not an independent prognostic factor in multivariate analysis (*p* = 0.221) (Table 3).

## 4. Discussion

Recently, various immunotherapies have been investigated in human malignancies, and several breakthrough approaches, which target the innate immune system, have been discovered [27]. Macrophages are important for an innate immune response and CD47 has been identified as a key macrophage checkpoint [28]. CD47 is a transmembrane protein which delivers a don’t-eat-me signal through SIPRα, which is expressed on the surface of macrophages [12]. The overexpression of CD47 has been reported in various types of cancers, such as non-small cell lung cancer, breast cancer, stomach cancer, and non-Hodgkin lymphoma and has been associated with an adverse prognosis [13,14,15,16]. After the role of CD47 in the process of tumor cell evasion from the immune system had been disclosed, targeting CD47 has become a novel strategy for treatment, and various anti-CD47 antibodies have been reported, such as Hu5F9-G4, ZF1, B6H12 antibodies, etc. [15,29,30]. Many CD47-targeted drugs have entered clinical trials, and promising results have been obtained [31].

CD47 is overexpressed in a majority of gastrointestinal tumors, and CD47 overexpression usually predicts an adverse prognosis [32]. Lascorz et al. revealed an association between an intronic single-nucleotide polymorphism of CD47 and CRA patient survival [33]. They reported that an increased CD47 expression was associated with distant metastasis and a poorer patient outcome. Zhang et al. focused on the role of TAM with the M2 phenotype [34]. They showed that the number of M2 macrophages in CRA tissues was elevated, which coincided with an increase in CD47 and SIRPα expression in these macrophages. They also revealed that the tumor cell migration and metastatic potential were increased in the CD47-induced TAM-rich microenvironment. Fujiwara-Tani et al. investigated the immunohistochemical expression of CD47 in CRA tissues and compared it with CD44 expression [35]. They demonstrated that the overexpression of CD47 was correlated with distant metastasis and that a high CD47 expression was associated with a shorter disease-free survival than was a low CD47 expression, in stage III patients. The authors also suggested that CD47 promotes epithelial-mesenchymal transition and that CD47 is involved in resistance to PD-1/PD-L1 inhibitors.

In this study, CD47 expression was examined via immunohistochemical analysis of human CRA tissues and correlated with various clinicopathologic factors. CD47 expression was observed in a minority (16.2%) of CRA tissues and was significantly correlated with adverse clinicopathologic features, including lymphatic invasion, perineural invasion, tumor budding, the pN stage, and the AJCC stage. In survival analyses, a positive CD47 expression was associated with a shorter RFS in all cases. These results are consistent with findings from previous CD47 studies in CRA. MSI-high CRAs elicit a strong anti-tumoral immune response in the host and, therefore, use different strategies to evade the immune system [36]. However, a survival difference was not observed in MSI-based subgroups in this study. This may be attributed to the fact that a small number of patients did not fully represent the clinical properties of the MSI molecular state. Further investigations with a large-scale cohort are needed to clarify the exact significance of CD47 expression in MSI-high CRA.

In survival analyses, positive CD47 expression was associated with a shorter RFS. However, it was not an independent prognostic factor in multivariate analysis. This may come from its association with adverse clinicopathologic features, especially the AJCC cancer stage. High CD47 expression was identified in the advanced-stage group (20.0%) more than the early-stage group (10.9%). The current eighth AJCC stage is determined by invasion depth (pT stage), lymph node metastasis (pN stage), and distant metastasis (pM stage) [23]. Among these, the pN stage (N0 vs. N1 or N2) is an important factor in distinguishing AJCC stage II from AJCC stage III. According to dichotomous analysis with clincopathologic factors (Table 2), high CD47 expression has a significant association with the pN stage as well as lymphatic invasion, which suggests that the permeability of the tumor may result in more frequent positive CD47 expression in AJCC stage III. In addition, the role of the immune environment in tumor progression has been emphasized, and many studies have focused on the immune-suppressive effects of the CD47-SIRPα axis, which is mediated by cancer cells and macrophages [10,37]. Immune evasion is important for progress to the advanced stage, and some studies have reported that higher tumor-associated macrophage count correlates with more advanced stages in CRA [37]. This may suggest that the don’t-eat-me signal, CD47, which prevents phagocytic activity, also increases in the advanced stage. Further studies are needed to evaluate the prognostic role of CD47 as an independent factor, in which other prognostic factors, such as tumor stage, are excluded.

No study has, thus far, investigated the prognostic role of CD47 according to treatment modalities in a large number of patients with CRA. Fujiwara-Tani et al. reported two nivolumab-refractory cases with a high CD47/CD44 expression and suggested that PD-1 inhibitor resistance and CD47 expression could be linked [35]. In ovarian cancer, Brightwell et al. reported that patients with a low CD47 expression showed a better response to standard therapy and demonstrated an improved overall survival [38]. In hepatocellular carcinoma, Lo et al. demonstrated that NF-κB-mediated up-regulation of CD47 promoted sorafenib resistance, and they proposed a CD47 targeted therapy in combination with sorafenib as a novel therapeutic regimen for hepatocellular carcinoma [39]. These results from other studies demonstrate that CD47 expression may be an immunologic shield to conventional adjuvant treatment and can be considered for targeted therapies. In our study, we revealed that CD47 expression correlated with shorter RFS in patients who did not receive adjuvant treatment. These findings suggest that CD47 could be used as a predictor of the recurrence risk in patients who did not receive additional treatment and that CD47 could be a candidate for targeted therapy in patients who do not receive adjuvant therapy. We additionally subdivided the patients into subgroups according to the chemotherapy and radiation treatment regimens. However, additional survival analyses according to the treatment options showed no significant survival differences in the patient outcome.

Although this study revealed that CD47 expression is associated with adverse clinicopathologic parameters and poor patient outcomes in CRA, it has several limitations. First, we retrospectively collected the cohort data, and selection bias was not completely eliminated. Second, we did not suggest a standard cut-off score for CD47 expression to predict the anti-CD47 treatment response and patient outcome. According to a meta-analysis report of CD47 expression, various cut-off values have been reported [40]. Further studies using various cut-off values in clinical trials are needed to validate the standard cut-off score of CD47 expression. Third, only one method using immunohistochemistry was used to investigate CD47 expression. Other techniques that use PCR or western blot or flow cytometry to detect CD47 proteins in tumor tissue and functional studies revealing association of tumor invasiveness with CD47 are required.

In conclusion, we investigated CD47 expression in 328 CRA patients. A positive CD47 expression was observed in 16.2% of the total CRA tissues and was significantly associated with adverse clinicopathologic features and worse patient outcomes, especially in CRA patients who did not receive adjuvant treatment.

## Figures and Tables

**Table 1:** Baseline characteristics of colorectal adenocarcinoma patients.

Factors	Value (%)
Number of patients	328 (100%)
Mean age at surgery (years)	63.6 (±11.19)
Median survival (months)	127 (1−182)
Sex	
Male	202 (61.6%)
Female	126 (38.4%)
Histologic grade	
Grade 1	17 (5.2%)
Grade 2	159 (48.5%)
Grade 3	133 (40.5%)
Grade 4	19 (5.8%)
Location	
Cecum & ascending	60 (18.3%)
Transverse	20 (6.1%)
Descending & rectosigmoid	248 (75.6%)
pT stage	
1	17 (5.2%)
2	33 (10.1%)
3	212 (64.6%)
4	66 (20.1%)
pN stage	
0	140 (42.7%)
1	88 (26.8%)
2	100 (30.5%)
M stage	
0	305 (93.0%)
1	23 (7.0%)
AJCC stage	
I	36 (11.0%)
II	102 (31.1%)
III	167 (50.9%)
IV	23 (7.0%)
Microsatellite instability (MSI) status	
Non-MSI-high	303 (92.4%)
MSI-high	25 (7.6%)
Treatment	
Surgery	173 (52.7%)
Surgery + Adjuvant therapy	155 (47.3%)

**Table 2:** Correlation between CD47 expression and clinicopathologic factors in colorectal adenocarcinoma.

Clinicopathologic Factors	*n*	CD47 Expression		*p* Value
		Negative (%) (*n* = 275)	Positive (%) (*n* = 53)	
Tumor size				0.642 *
Mean	328	5.13 (±2.12)	5.28 (±2.05)	
Gross type				0.940
Polypoid	31	26 (83.9%)	5 (16.1%)	
Ulcerofungating	143	121 (84.6%)	22 (15.4%)	
Ulceroinfiltrative	154	128 (83.1%)	26 (16.9%)	
Histologic grade				0.301
Grade 1 & Grade2	176	151 (85.8%)	25 (14.2%)	
Grade 3 & Grade 4	152	124 (81.6%)	28 (18.4%)	
Lymphatic invasion				0.018
Not identified	141	126 (89.4%)	15 (10.6%)	
Identified	187	149 (79.7%)	38 (20.3%)	
Vascular invasion				0.659
Not identified	267	225 (84.3%)	42 (15.7%)	
Identified	61	50 (82.0%)	11 (18.0%)	
Perineural invasion				0.024
Not identified	164	145 (88.4%)	19 (11.6%)	
Identified	164	130 (79.3%)	34 (20.7%)	
Tumor deposit				0.488
Not identified	265	224 (84.5%)	41 (15.5%)	
Identified	63	51 (81.0%)	12 (19.0%)	
Tumor budding				0.009
Low grade	195	172 (88.2%)	23 (11.8%)	
High grade	133	103 (77.4%)	30 (22.6%)	
pT stage				0.099
T1,T2	49	45 (91.8%)	4 (8.2%)	
T3,T4	279	230 (82.4%)	49 (17.6%)	
pN stage				0.022
N0	140	125 (89.3%)	15 (10.7%)	
N1 & N2	188	150 (79.8%)	38 (20.2%)	
Extranodal tumor extension				0.210
Not identified	106	88 (83.0%)	18 (17.0%)	
Identified	82	62 (75.6%)	20 (24.4%)	
Distant metastasis				0.180
M0	305	258 (84.6%)	47 (15.4%)	
M1a & M1b	23	17 (73.9%)	6 (26.1%)	
AJCC stage				0.027
I & II	138	123 (89.1%)	15 (10.9%)	
III & IV	190	152 (80.0%)	38 (20.0%)	
MSI status				0.268
Non-MSI-high	303	256 (84.5%)	47 (15.5%)	
MSI-high	25	19 (76.0%)	6 (24.0%)	

* Mann–Whitney test.

**Table 3:** Univariate Cox regression analysis of prognostic factors for patient survival in colorectal adenocarcinoma.

Variable	Univariate Analysis		Multivariate Analysis	
	HR (95% CI)	*p* Value	HR (95% CI)	*p* Value
Recurrence-free survival				
CD47 expression (positive vs. negative)	1.792 (1.014–3.084)	0.035	1.406 (0.815–2.428)	0.221
AJCC stage (I & II vs. III & IV)	3.356 (1.901–5.925)	<0.001	1.762 (0.930–3.338)	0.083
MSI status (non-MSI-high vs. MSI-high)	1.155 (0.466–2.863)	0.756	1.158 (0.464–2.893)	0.753
Treatment (surgery only vs. surgery + Adj *)	4.660 (2.675–8.120)	<0.001	3.474 (1.863–6.476)	<0.001
Cancer-specific survival				
CD47 expression (positive vs. negative)	1.176 (0.770–1.794)	0.453	1.092 (0.713–1.672)	0.686
AJCC stage (I & II vs. III & IV)	1.796 (1.277–2.526)	0.001	1.848 (1.247–2.737)	0.002
MSI status (non-MSI-high vs. MSI-high)	1.175 (0.618–2.231)	0.623	1.050 (0.550–2.004)	0.882
Treatment (surgery only vs. surgery + Adj *)	1.273 (0.925–1.752)	0.138	1.075 (0.745–1.551)	0.699

HR: hazard ratio; CI: confidence interval; Adj *: adjuvant therapy.

## References

[1] Ferlay J., Colombet M., Soerjomataram I., Mathers C., Parkin D.M., Piñeros M., Znaor A., Bray F. (2019). Estimating the global cancer incidence and mortality in 2018: GLOBOCAN sources and methods. Int. J. Cancer.

[2] Cronin K.A., Lake A.J., Scott S., Sherman R.L., Noone A.M., Howlader N., Henley S.J., Anderson R.N., Firth A.U., Ma J. (2018). Annual Report to the Nation on the Status of Cancer, part I: National cancer statistics. Cancer.

[3] Jackson S.E., Chester J.D. (2015). Personalised cancer medicine. Int. J. Cancer.

[4] Oh H.H., Joo Y.E. (2020). Novel biomarkers for the diagnosis and prognosis of colorectal cancer. Intest. Res..

[5] Vinay D.S., Ryan E.P., Pawelec G., Talib W.H., Stagg J., Elkord E., Lichtor T., Decker W.K., Whelan R.L., Kumara H. (2015). Immune evasion in cancer: Mechanistic basis and therapeutic strategies. Semin. Cancer Biol..

[6] Long A.G., Lundsmith E.T., Hamilton K.E. (2017). Inflammation and Colorectal Cancer. Curr. Colorectal Cancer Rep..

[7] Stidham R.W., Higgins P.D.R. (2018). Colorectal Cancer in Inflammatory Bowel Disease. Clin. Colon Rectal Surg..

[8] Tong B., Wang M. (2018). CD47 is a novel potent immunotherapy target in human malignancies: Current studies and future promises. Future Oncol..

[9] Mantovani A., Marchesi F., Malesci A., Laghi L., Allavena P. (2017). Tumour-associated macrophages as treatment targets in oncology. Nat. Rev. Clin. Oncol..

[10] Willingham S.B., Volkmer J.P., Gentles A.J., Sahoo D., Dalerba P., Mitra S.S., Wang J., Contreras-Trujillo H., Martin R., Cohen J.D. (2012). The CD47-signal regulatory protein alpha (SIRPa) interaction is a therapeutic target for human solid tumors. Proc. Natl. Acad. Sci. USA.

[11] Matlung H.L., Szilagyi K., Barclay N.A., van den Berg T.K. (2017). The CD47-SIRPα signaling axis as an innate immune checkpoint in cancer. Immunol. Rev..

[12] Liu X., Kwon H., Li Z., Fu Y.X. (2017). Is CD47 an innate immune checkpoint for tumor evasion?. J. Hematol. Oncol..

[13] Barrera L., Montes-Servín E., Hernandez-Martinez J.M., García-Vicente M., Montes-Servín E., Herrera-Martínez M., Crispín J.C., Borbolla-Escoboza J.R., Arrieta O. (2017). CD47 overexpression is associated with decreased neutrophil apoptosis/phagocytosis and poor prognosis in non-small-cell lung cancer patients. Br. J. Cancer.

[14] Baccelli I., Stenzinger A., Vogel V., Pfitzner B.M., Klein C., Wallwiener M., Scharpff M., Saini M., Holland-Letz T., Sinn H.P. (2014). Co-expression of MET and CD47 is a novel prognosticator for survival of luminal breast cancer patients. Oncotarget.

[15] Yoshida K., Tsujimoto H., Matsumura K., Kinoshita M., Takahata R., Matsumoto Y., Hiraki S., Ono S., Seki S., Yamamoto J. (2015). CD47 is an adverse prognostic factor and a therapeutic target in gastric cancer. Cancer Med..

[16] Chao M.P., Tang C., Pachynski R.K., Chin R., Majeti R., Weissman I.L. (2011). Extranodal dissemination of non-Hodgkin lymphoma requires CD47 and is inhibited by anti-CD47 antibody therapy. Blood.

[17] Chao M.P., Alizadeh A.A., Tang C., Myklebust J.H., Varghese B., Gill S., Jan M., Cha A.C., Chan C.K., Tan B.T. (2010). Anti-CD47 antibody synergizes with rituximab to promote phagocytosis and eradicate non-Hodgkin lymphoma. Cell.

[18] Gholamin S., Mitra S.S., Feroze A.H., Liu J., Kahn S.A., Zhang M., Esparza R., Richard C., Ramaswamy V., Remke M. (2017). Disrupting the CD47-SIRPα anti-phagocytic axis by a humanized anti-CD47 antibody is an efficacious treatment for malignant pediatric brain tumors. Sci. Transl. Med..

[19] Liu L., Zhang L., Yang L., Li H., Li R., Yu J., Yang L., Wei F., Yan C., Sun Q. (2017). Anti-CD47 Antibody as a Targeted Therapeutic Agent for Human Lung Cancer and Cancer Stem Cells. Front. Immunol..

[20] Lo J., Lau E.Y., So F.T., Lu P., Chan V.S., Cheung V.C., Ching R.H., Cheng B.Y., Ma M.K., Ng I.O. (2016). Anti-CD47 antibody suppresses tumour growth and augments the effect of chemotherapy treatment in hepatocellular carcinoma. Liver Int. Off. J. Int. Assoc. Study Liver.

[21] Xiao Z., Chung H., Banan B., Manning P.T., Ott K.C., Lin S., Capoccia B.J., Subramanian V., Hiebsch R.R., Upadhya G.A. (2015). Antibody mediated therapy targeting CD47 inhibits tumor progression of hepatocellular carcinoma. Cancer Lett..

[22] Weiskopf K. (2017). Cancer immunotherapy targeting the CD47/SIRPα axis. Eur. J. Cancer.

[23] Amin M., Edge S., Greene R., Byrd D., Brooklnad R., Washington M., Gershenwald J., Compton C., Hess K., Sullivan D. (2017). AJCC Cancer Staging Manual.

[24] Washington M.K., Berlin J., Branton P., Burgart L.J., Carter D.K., Fitzgibbons P.L., Halling K., Frankel W., Jessup J., Kakar S. (2009). Protocol for the examination of specimens from patients with primary carcinoma of the colon and rectum. Arch. Pathol. Lab. Med..

[25] Kim H., Rehman A., Chung Y., Yi K., Wi Y.C., Kim Y., Jang K., Jang S.M., Paik S.S. (2016). Clinicopathologic Significance of Extranodal Tumor Extension in Colorectal Adenocarcinoma with Regional Lymph Node Metastasis. Gastroenterol. Res. Pract..

[26] Boland C.R., Thibodeau S.N., Hamilton S.R., Sidransky D., Eshleman J.R., Burt R.W., Meltzer S.J., Rodriguez-Bigas M.A., Fodde R., Ranzani G.N. (1998). A National Cancer Institute Workshop on Microsatellite Instability for cancer detection and familial predisposition: Development of international criteria for the determination of microsatellite instability in colorectal cancer. Cancer Res..

[27] Chao M.P., Takimoto C.H., Feng D.D., McKenna K., Gip P., Liu J., Volkmer J.P., Weissman I.L., Majeti R. (2019). Therapeutic Targeting of the Macrophage Immune Checkpoint CD47 in Myeloid Malignancies. Front. Oncol..

[28] Lian S., Xie X., Lu Y., Jia L. (2019). Checkpoint CD47 Function on Tumor Metastasis and Immune Therapy. Oncotargets Ther..

[29] Liu J., Wang L., Zhao F., Tseng S., Narayanan C., Shura L., Willingham S., Howard M., Prohaska S., Volkmer J. (2015). Pre-Clinical Development of a Humanized Anti-CD47 Antibody with Anti-Cancer Therapeutic Potential. PLoS ONE.

[30] Zeng D., Sun Q., Chen A., Fan J., Yang X., Xu L., Du P., Qiu W., Zhang W., Wang S. (2016). A fully human anti-CD47 blocking antibody with therapeutic potential for cancer. Oncotarget.

[31] Zhang W., Huang Q., Xiao W., Zhao Y., Pi J., Xu H., Zhao H., Xu J., Evans C.E., Jin H. (2020). Advances in Anti-Tumor Treatments Targeting the CD47/SIRPα Axis. Front. Immunol..

[32] Tzatzarakis E., Hissa B., Reissfelder C., Schölch S. (2019). The overall potential of CD47 in cancer immunotherapy: With a focus on gastrointestinal tumors. Expert Rev. Anticancer Ther..

[33] Lascorz J., Bevier M., Schönfels W.V., Kalthoff H., Aselmann H., Beckmann J., Egberts J., Buch S., Becker T., Schreiber S. (2013). Association study identifying polymorphisms in CD47 and other extracellular matrix pathway genes as putative prognostic markers for colorectal cancer. Int. J. Colorectal Dis..

[34] Zhang Y., Sime W., Juhas M., Sjölander A. (2013). Crosstalk between colon cancer cells and macrophages via inflammatory mediators and CD47 promotes tumour cell migration. Eur. J. Cancer.

[35] Fujiwara-Tani R., Sasaki T., Ohmori H., Luo Y., Goto K., Nishiguchi Y., Mori S., Nakashima C., Mori T., Miyagawa Y. (2019). Concurrent Expression of CD47 and CD44 in Colorectal Cancer Promotes Malignancy. Pathobiol. J. Immunopathol. Mol. Cell. Biol..

[36] Kloor M., Michel S., von Knebel Doeberitz M. (2010). Immune evasion of microsatellite unstable colorectal cancers. Int. J. Cancer.

[37] Kang J.C., Chen J.S., Lee C.H., Chang J.J., Shieh Y.S. (2010). Intratumoral macrophage counts correlate with tumor progression in colorectal cancer. J. Surg. Oncol..

[38] Brightwell R.M., Grzankowski K.S., Lele S., Eng K., Arshad M., Chen H., Odunsi K. (2016). The CD47 “don’t eat me signal” is highly expressed in human ovarian cancer. Gynecol. Oncol..

[39] Lo J., Lau E.Y., Ching R.H., Cheng B.Y., Ma M.K., Ng I.O., Lee T.K. (2015). Nuclear factor kappa B-mediated CD47 up-regulation promotes sorafenib resistance and its blockade synergizes the effect of sorafenib in hepatocellular carcinoma in mice. Hepatology.

[40] Zhao H.-J., Pa F., Shi Y.-C., Luo X., Ren R.-R., Peng L.-H., Yang Y.-S. (2018). Prognostic significance of CD47 in human malignancies: A systematic review and meta-analysis. Transl. Cancer Res..

